# Beyond diagnosis: symptom patterns across complex PTSD and borderline personality disorder

**DOI:** 10.3389/fpsyt.2025.1668821

**Published:** 2025-10-30

**Authors:** Joe J. Simon, Kristin Spiegler, Kadiatou Coulibaly, Marion A. Stopyra, Hans-Christoph Friederich, Oliver Gruber, Christoph Nikendei

**Affiliations:** ^1^ Department of General Internal Medicine and Psychosomatics, Centre for Psychosocial Medicine, University Hospital Heidelberg, Heidelberg, Germany; ^2^ DZPG - German Centre for Mental Health (Partner Site Heidelberg/Mannheim/Ulm), Heidelberg, Germany; ^3^ Department of Clinical Psychology and Psychotherapy, Institute of Psychology, Heidelberg University, Heidelberg, Germany; ^4^ Department of Clinical Psychology and Psychotherapy, University of Ulm,Ulm, Germany; ^5^ Department of General Psychiatry, Centre for Psychosocial Medicine, University Hospital Heidelberg, Heidelberg, Germany

**Keywords:** complex post-traumatic stress disorder, borderline personality disorder, trauma, dissociation, affective symptoms, functional impairment

## Abstract

**Background:**

Complex post-traumatic stress disorder (cPTSD) and borderline personality disorder (BPD) share overlapping clinical features, complicating accurate diagnosis and treatment. This overlap has fueled ongoing discussions regarding whether cPTSD and BPD should be considered as distinct diagnostic categories. To contribute to this debate, the current study aimed to clarify symptomatic similarities and differences between cPTSD and BPD using psychometric assessments.

**Methods:**

97 female participants were recruited, including 34 patients with cPTSD, 25 with BPD and 38 healthy controls. All participants completed a battery of validated psychometric instruments assessing depression, anxiety, difficulties in emotion regulation, psychodynamic dysfunction, guilt-related distress, health-related functioning, as well as trauma-related symptoms and borderline personality traits. Differences between groups were analyzed using one-way ANOVAs, followed by *post-hoc* comparisons.

**Results:**

Patients with cPTSD reported significantly higher levels of trauma exposure, posttraumatic symptom severity, dissociative symptoms, affective symptoms and functional impairment compared to both BPD patients and healthy controls. In contrast, no significant differences were found between cPTSD and BPD in borderline symptom severity, anxiety, difficulties in emotion regulation, guilt-related distress and psychodynamic dysfunction.

**Conclusions:**

The results highlight trauma-related symptoms as key differentiators between cPTSD and BPD, supporting the conceptualization of cPTSD as a distinct disorder. However, as ICD-11 specific diagnostic instruments were not applied, the findings should be interpreted with appropriate caution.

## 1 Introduction

Posttraumatic stress disorder (PTSD) is a psychiatric disorder that develops in response to exposure to a traumatic event or series of events, characterized by re-experiencing, avoidance, and a persistent sense of current threat. According to epidemiological data, an estimated 3.9% of the global population experiences PTSD during their lifetime (1). While PTSD is typically associated with single-incident trauma, individuals exposed to prolonged or repeated interpersonal trauma may present with a broader symptom profile. To account for these cases, the 11th revision of the International Classification of Diseases (ICD-11) introduced complex PTSD (cPTSD) as a distinct diagnosis (2). cPTSD includes the core PTSD symptoms along with persistent disturbances in self-organization (DSO), which are defined as affect dysregulation, a negative self-concept, and difficulties in interpersonal relationships (3, 4). These features reflect impairments in personality functioning, as they interfere with self-regulation and relational capacities.

Borderline personality disorder (BPD) is another clinical condition commonly associated with trauma exposure and impairments in self- and interpersonal functioning. It is characterized by a pervasive pattern of instability in affect, self-image, relationships, and impulse control (5). Although complex PTSD (cPTSD) and BPD share several features, such as emotion dysregulation and relational difficulties (6), they are classified as separate disorders in the ICD-11 and differ in terms of symptom profiles, developmental pathways, and diagnostic criteria (7). The considerable symptom overlap between cPTSD and BPD has raised ongoing questions about their diagnostic distinction (8–10). This overlap can make clinical differentiation challenging and may contribute to the frequent co-occurrence of both diagnoses. Indeed, studies have found that approximately half of individuals diagnosed with cPTSD also meet criteria for BPD (6; Martin 11), which may reflect shared features that complicate the diagnostic process rather than simple comorbidity (12). This perspective also aligns with dimensional models of psychopathology, which conceptualize disorders as varying expressions of shared underlying traits rather than strictly separate categories (13). While some authors have argued for a reconceptualization of cPTSD as a combination of PTSD and BPD (8), or even questioned the need to separate the two disorders (14), most empirical research supports maintaining them as distinct diagnostic entities, based on key differences in symptom expression, etiology, and treatment needs (3, 8, 11, 12, 15). For example, cPTSD centers trauma as the core etiological factor, whereas emotional neglect has been identified as a predominant risk factor in the development of BPD (16).

In a recent review of studies investigating the association between BPD and cPTSD, Karatzias et al. (7) outlined critical differences in diagnostic criteria that could help in adequately distinguishing the two disorders. First, although a history of traumatic life events is common in BPD, unlike in cPTSD, it is not a diagnostic criterion (17–19). Second, suicidal and self-injurious behaviors are common in BPD, but occur less frequently in cPTSD (20). Third, difficulties in affect regulation in cPTSD are ego-dystonic and characterized by prolonged states of distress and heightened reactivity to interpersonal stress, whereas in BPD, affect dysregulation and unstable mood appear ego-syntonic and relatively stable over time. Fourth, BPD is associated with an unstable sense of self, fluctuating between extreme self-valuation and self-devaluation, whereas cPTSD is marked by persistently negative self-beliefs and pervasive feelings of guilt, shame, and worthlessness (4). Lastly, while interpersonal difficulties in BPD involve volatile patterns of interaction, and frantic efforts to avoid abandonment, due to hypervigilance or increased sensitivity to perceived harm from others, cPTSD is marked by patterns of social avoidance and isolation and associated persistent interpersonal distrust.

The common conclusion of previous studies investigating symptomatic differences is that despite the significant symptomatic overlap in clinical features, empirical evidence suggests that the two disorders are distinct entities. For example, statistical analyses of symptom patterns have shown that BPD can be distinguished from cPTSD by differences in fear of abandonment, impairment in interpersonal relationships, impulsivity and an unstable sense of self (3). As noted by Ford and Courtois, (6) cPTSD may be conceptualized as a maladaptive stress response that progresses from hypervigilance as a characteristic of PTSD, toward emotional and relational withdrawal. BPD, on the other hand, might develop as a fight response where, instead of a hypervigilance or emotional detachment, a diminished sense of self is coupled with impulsive, disorganized, and conflict-driven behavior in interpersonal interactions (6).

Nevertheless, despite these findings, a clear and standardized distinction remains challenging, highlighting the need for further research. Misdiagnosis due to symptom overlap can significantly compromise therapeutic outcomes, as treatment strategies for BPD and cPTSD differ substantially (6, 8, 11). Therefore, the present study aimed to disentangle symptomatic similarities and differences between cPTSD and BPD. Our goal was to improve the understanding of clinical features associated with each disorder, to validate previous empirical distinctions, and to highlight areas of symptomatic overlap. To this end, we used a comprehensive array of psychometric instruments to assess trauma and borderline-related symptom expression and general psychopathology in a group of patients diagnosed with BPD and cPTSD.

## 2 Methods

### 2.1 Participants

A total of 97 women (sex assigned at birth, gender identity not systematically assessed) were included in the present study: 34 patients with cPTSD, 25 patients with BPD and 38 healthy controls (HC). The restriction to women was chosen because the majority of patients treated for cPTSD and BPD in our setting are women, and it also ensured consistency for the fMRI companion study (21). *A priori* considerations based on effect sizes reported in comparable fMRI paradigms (d ≈ 1.6–1.7; 22, 23). and methodological recommendations (24, 25) suggested that a target of approximately 30 participants per group would provide sufficient power, although clinical symptom contrasts may be associated with smaller effect sizes and thus lower power. Clinical participants were recruited from our outpatient clinics and psychosomatic wards (Department of General Internal Medicine and Psychosomatics, Centre for Psychosocial Medicine, University Hospital Heidelberg, Heidelberg, Germany), while HC were recruited via public advertisements. All participants were recruited between June 2020 and July 2022 and provided written informed consent according to the Declaration of Helsinki. Participants were eligible for inclusion if they met diagnostic criteria for cPTSD or BPD as defined with the 11^th^ edition of the International Classification of Diseases, were female and were aged 18 years or older. Exclusion criteria were severe psychiatric comorbidities, active substance use disorder or psychotropic medication use other than stable antidepressant treatment. The present study was part of a larger fMRI investigation, examining emotional reactivity and neural reward processing in patients with cPTSD, as reported elsewhere (21). Therefore, additional inclusion criteria were right-handedness as well as normal or corrected-to-normal vision, and additional exclusion criteria included a history of head injury or surgery, neurological disorders, smoking, fMRI contraindications, or pregnancy. For the MRI study, 7 patients with cPTSD, 4 patients with BPD, and 1 healthy control were excluded due to poor task performance or excessive head movement during scanning. However, all participants were included in the present study. The ethics committee of the Medical Faculty of Heidelberg approved this study (file no. S-260/2018). For the participation, a fixed amount of 40 EUR was paid with the opportunity to win an additional amount of 30 EUR in the experimental task during the fMRI study (21). Details on psychotropic medication by group are reported in **Supplementary Table S1**.

### 2.2 Study procedure

The study took place in individual, single sessions between 10:00 AM and 2:00 PM. In both patient groups and healthy controls, psychiatric diagnoses were established using the Structured Clinical Interview for DSM IV (SCID I and II; 26). Healthy controls were screened for lifetime psychiatric disorders with the SCID and patients with cPTSD and BPD were included if they fulfilled the diagnostic criteria as defined in the 11th edition of the International Classification of Diseases of the World Health Organization. SCID results were compared with available clinical records, and if both sources agreed, the patient was included. In cases of discrepancy, a case by case decision was reached in consensus, and final diagnostic validation was carried out by JJS and CN. Following the interview, participants completed a battery of psychometric scales assessing trauma and borderline-related symptoms and functional impairment (see below). Although all patients were included based on ICD-11 criteria for cPTSD and BPD, symptom assessment relied on DSM-based instruments. This decision ensured use of psychometrically robust and widely applied measures that facilitate comparability with prior research, but it also reflects a hybrid approach in which ICD-11 diagnoses were operationalized using DSM-oriented tools. Furthermore, demographic information was collected including age, highest educational attainment and vocational qualification. To estimate premorbid intelligence, participants completed the Multiple-Choice Vocabulary Test (MWT-B; 27), a widely used measure of crystallized verbal intelligence.

### 2.3 Psychometric assessment

All psychometric instruments employed in this study were administered in their German versions. To assess trauma-related symptoms, all participants completed the Trauma Symptom Inventory-2 (TSI-2; 28, 29) and the Posttraumatic Diagnostic Scale (PDS; 30, 31). To assess symptoms associated with cPTSD, all participants completed the Screening for Complex Post-Traumatic Stress Disorder Scale (skPTBS; 32, 33). Adverse childhood experiences were assessed with the Adverse Childhood Experiences Questionnaire (ACE-D; 34, 35) and life-time experiences of traumatic events were assessed with the Life Events Checklist for DSM-5 (LEC-5; 36, 37). Symptoms of dissociation were assessed with the German version of the Dissociative Experiences Scale (FDS-20; 38, 39).

To assess BPD-related symptoms, we employed the Borderline Symptom List-2 (BSL-23; 40, 41).

To assess general psychopathology, we used scales assessing symptoms of depression: Beck Depression Inventory (BDI-II; 42, 43) and Patient Health Questionnaire (PHQ-9; 44), to assess generalized anxiety disorder we used the Generalizied Anxiety Disorder Scale (GAD-7; 45) and health-related quality of life was assessed using the Short Form Health Survey (SF-12; 46, 47). Furthermore, we assessed difficulties in emotion regulation using the Difficulties in Emotion Regulation Scale (DERS; 48, 49), hedonic experience using the Temporal Experience of Pleasure Scale (TEPS; 50, 51), interpersonal guilt using the German short version of the Interpersonal Guilt Questionnaire (FIS; 52, 53), and structural abilities within the Operationalized Psychodynamic Diagnosis framework (OPD Structure Questionnaire, OPD-SQ; 54). The OPD-SQ captures psychodynamic dysfunctions, which are defined as impairments in structural abilities of the self and in interpersonal functioning. These structural abilities encompass self-perception, the capacity for interpersonal contact, and the internalization of relationship models. The OPD-SQ short form provides both a total score and specific subscales that reflect these domains.

### 2.4 Statistical analysis

Data were analyzed using R, (RRID: SCR_004042; 55). One-way analyses of variance (ANOVAs) with group as the between-subjects factor were used to examine psychometric differences between groups. Primary analyses were conducted using analysis of covariance (ANCOVA) with group as the between-subjects factor and age and medication status as covariates. To account for correlations among outcomes, we additionally implemented within-domain multivariate analyses of covariance (MANCOVAs) for the TSI symptom scales, TSI functional scales, DERS subscales, and OPD subscales. For all ANOVAs, eta-squared (η²) was used as the measure of effect size. For interpretability, standardized mean differences (Hedges g) with 95% confidence intervals were calculated for contrasts between cPTSD and BPD. Significant results were further examined using *post-hoc* tests, adjusted for multiple comparisons. Tukey HSD was used when variances were homogeneous, whereas Games–Howell was used for nonhomogeneous variances. Assumption checks (Shapiro–Wilk tests within groups and Levene’s tests for homogeneity) and a group-wise missingness summary are reported in the **Supplementary Tables S2**-**S4**. Missing data were minimal (≤ 5% per variable across groups) and handled by listwise deletion, which explains the minor variation in sample sizes and degrees of freedom across analyses. All p values were adjusted for multiple testing using the Benjamini Hochberg false discovery rate procedure. We report results significant at P < 0.05 corrected for multiple comparisons. As an additional robustness check, we attempted propensity score matching on age for the cPTSD and BPD groups. However, due to limited sample size and insufficient overlap, a stable matched sample could not be retained. Thus, the main results rely on ANCOVAs with age and medication as covariates. As an exploratory step, we implemented a cross-validated penalized logistic regression (LASSO) model using trauma-related scales to discriminate between cPTSD and BPD.

To facilitate interpretability, we additionally reported proportions of participants exceeding established clinical thresholds for all self-report measures where cut-offs are available. Specifically, cut-offs were applied for the BDI-II (56), PHQ-9 (57), GAD-7 (58), ACE-D (59), FDS-20 (60), and BSL-23 (61). For the LEC-5, we followed the standard approach (62), scoring each of the 17 traumatic event categories as endorsed if participants reported the event as “happened to me,” “witnessed it,” or “learned about it.” The total score therefore represents the number of distinct event categories experienced, with higher totals indicating greater cumulative trauma exposure. Observed score ranges are provided in **Supplementary Table S6**, and proportions above clinical cut-offs are summarized in **Supplementary Tables S7**-**S12**. Internal consistency (Cronbach’s alpha) was calculated for all self-report instruments and subscales where this was possible; results are reported in the **Supplementary Material** (**Supplementary Table S13**).

## 3 Results

### 3.1 Differences in sociodemographic characteristics

Demographic and cognitive data for the groups are presented in **Table 1**. A significant group difference was found for age, with the BPD group being significantly younger than both the cPTSD and control groups. No differences were found in education, vocational qualification or premorbid intelligence.

**Table 1:** Group comparisons for demographic measures.

Demographic variable	Groups			ANOVA		Significant *post-hoc* tests: cPTSD vs. BPD		
	cPTSD M (SD)	BPD M (SD)	Control M (SD)	F(df)	η²	Mean difference (cPTSD – BPD)	95% CI	n (cPTSD/BPD/HC)
Age	36.42 (11.98)	28.27 (7.23)	35.88 (11.07)	5.15 (2, 94)^***^	.099	- 8.14^*^	[-14.78, -1.50]	34/25/38
Educational attainment	5.80 (1.27)	5.76 (1.17)	6.34 (1.34)	2.23 (2,94)	.046			34/25/38
Vocational qualification	2.79 (2.39)	2.52 (1.64)	2.95 (1.29)	0.68 (2,94)	.014			34/25/38
Premorbid intelligence (MWT-B)	29.19 (4.00)	28.4 (2.66)	28.84 (3.98)	0.32 (2,92)	.007			32/25/38

η² = partial eta squared (effect size). Mean difference = Positive values indicate higher scores in the cPTSD group. CI = 95% confidence interval. ^***^ *p* <.001; ^*^ *p* <.05.

### 3.2 Trauma-related symptoms

**Figure 1:**
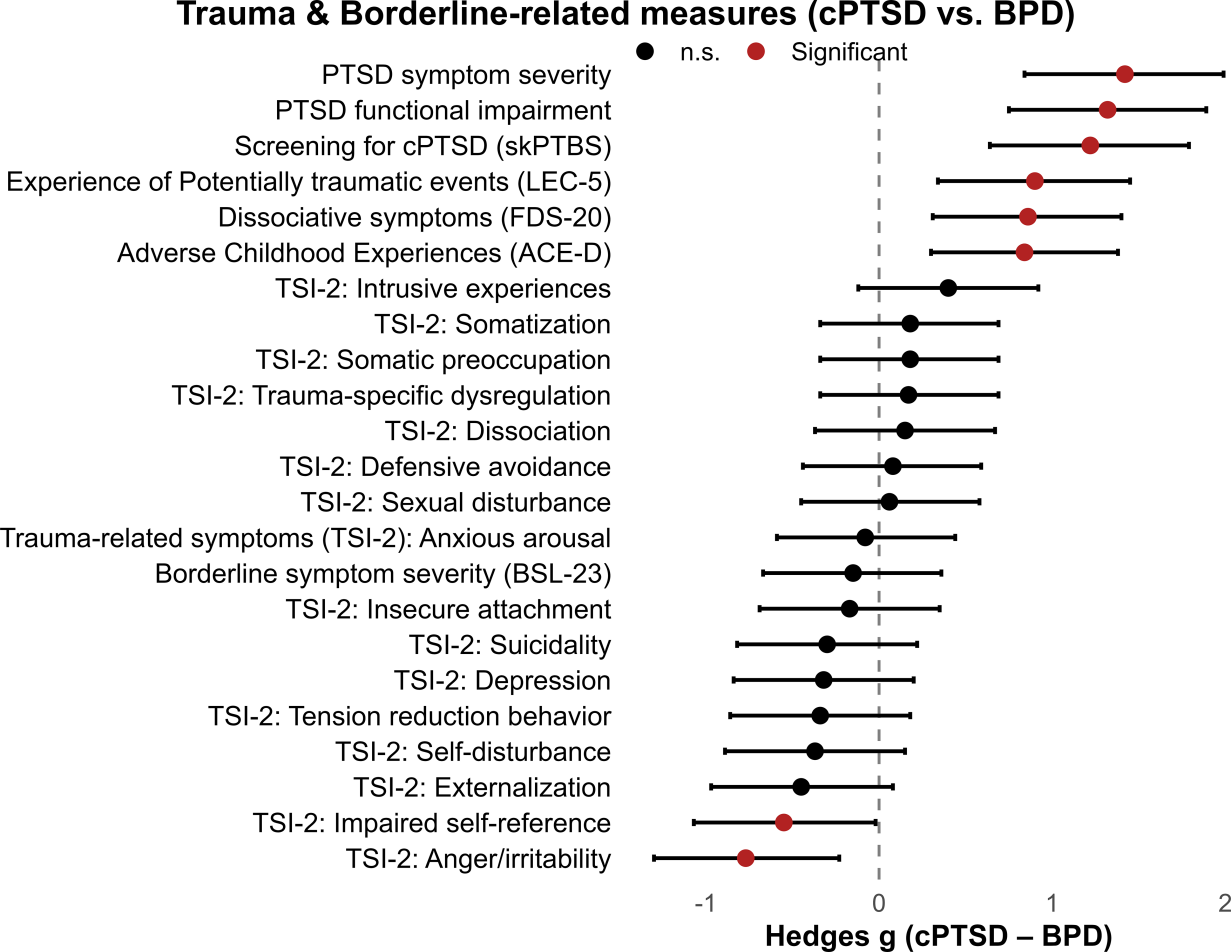
Trauma- and borderline-related measures in patients with cPTSD and BPD. Forest plot of standardized mean differences (Hedges g) with 95% confidence intervals comparing cPTSD and BPD groups across trauma-related and borderline symptom domains. Positive values indicate higher scores in the cPTSD group; negative values indicate higher scores in the BPD group.

We found significant group differences between patients and healthy controls across all trauma-related domains, with patients having increased scores throughout. Furthermore, except for one scale, we found that patients with cPTSD displayed higher scores in all trauma-related domains when compared to patients with BPD. Specifically, patients with cPTSD showed higher scores in the screening scale for cPTSD, adverse childhood experiences, exposure to potentially traumatic events, PTSD symptom severity, PTSD functional impairment and dissociative symptoms. For the TSI-2 scale, which assess different types of trauma-related symptoms, we found no differences between patient groups, except for the subscale assessing “anger and irritability”, with higher scores in the BPD group. These results were obtained from ANCOVAs adjusted for age and medication status. MANCOVAs confirmed significant multivariate group effects across TSI symptom and functional scales (all Wilks’ Λ < 0.44, p <.001). Please see **Table 2** for a detailed depiction of groups scores and group differences. Standardized mean differences between cPTSD and BPD across trauma- and borderline-related measures are shown in **Figure 1**.

**Table 2:** Group comparisons for trauma-related measures.

Questionnaire	Groups			ANCOVA		Significant *post-hoc* tests: cPTSD vs. BPD		
	cPTSD M (SD)	BPD M (SD)	Control M (SD)	F(df), p	η²	Hedges g	95% CI	n (cPTSD/BPD/HC)
Screening for cPTSD								
skPTBS: Total score	145.48 (30.03)	96.80 (49.58)	20.63 (28.51)	100.12 (df=2,89) p<0.001	0.692	1.22	[0.64, 1.79]	31/25/38
Adverse Childhood Experiences								
ACE-D	6.03 (2.68)	3.92 (2.24)	1.18 (1.67)	45.44 (df=2,92) p<0.001	0.497	0.84	[0.30, 1.38]	34/25/38
Potentially traumatic events								
LEC-5	55.31 (21.28)	35.04 (23.95)	5.79 (9.67)	44.21 (df=2,80) p<0.001	0.525	0.90	[0.34, 1.45]	32/24/29
PTSD symptom severity								
PDS: Severity	32.70 (10.91)	18.33 (8.83)	2.09 (4.30)	80.95 (df=2,92) p<0.001	0.638	1.42	[0.84, 1.99]	34/25/38
PTSD functional impairment								
PDS: Functional impairment	6.64 (1.98)	3.48 (3.12)	0.37 (1.09)	60.54 (df=2,92) p<0.001	0.568	1.32	[0.75, 1.89]	34/25/38
Dissociative symptoms								
FDS-20	31.31 (17.85)	17.64 (12.80)	1.88 (2.46)	38.32 (df=2,91) p<0.001	0.457	0.86	[0.31, 1.40]	33/25/38
Trauma-related symptoms								
TSI-2: Anxious arousal	14.97 (9.67)	15.60 (6.05)	4.95 (4.44)	18.43 (df=2,92) p<0.001	0.286			34/25/38
TSI-2: Depression	16.03 (10.80)	19.16 (7.99)	3.74 (4.82)	23.01 (df=2,92) p<0.001	0.333			34/25/38
TSI-2: Anger/irritability	8.91 (7.67)	14.56 (6.92)	3.53 (3.38)	19.63 (df=2,92) p<0.001	0.299	-0.77	[-1.30, -0.23]	34/25/38
TSI-2: Intrusive experiences	15.09 (10.20)	11.32 (7.98)	2.60 (3.86)	21.81 (df=2,92) p<0.001	0.322			34/25/38
TSI-2: Defensive avoidance	15.50 (11.08)	14.72 (8.22)	3.21 (5.11)	20.38 (df=2,92) p<0.001	0.307			34/25/38
TSI-2: Dissociation	10.91 (12.74)	9.36 (5.96)	1.05 (1.90)	8.98 (df=2,92) p=0.001	0.163			34/25/38
TSI-2: Somatic preoccupation	11.73 (9.09)	10.32 (6.08)	3.79 (3.73)	14.50 (df=2,92) p<0.001	0.240			34/25/38
TSI-2: Sexual disturbance	6.68 (6.46)	6.24 (7.26)	0.84 (2.24)	9.81 (df=2,92) p<0.001	0.176			34/25/38
TSI-2: Suicidality	5.59 (6.56)	7.68 (7.59)	0.42 (1.50)	6.48 (df=2,92) p=0.006	0.123			34/25/38
TSI-2: Insecure attachment	14.50 (9.78)	16.00 (6.88)	3.34 (4.21)	25.22 (df=2,92) p<0.001	0.354			34/25/38
TSI-2: Impaired self-reference	9.91 (8.55)	14.52 (8.22)	2.03 (4.55)	18.94 (df=2,92) p<0.001	0.292			34/25/38
TSI-2: Tension reduction behavior	7.74 (7.27)	10.28 (7.62)	1.00 (1.92)	13.32 (df=2,92) p<0.001	0.225			34/25/38
TSI-2: Self-disturbance	40.44 (27.53)	49.68 (20.51)	9.11 (12.44)	25.96 (df=2,92) p<0.001	0.361			34/25/38
TSI-2: Trauma-specific dysregulation	56.47 (36.90)	51.00 (22.27)	11.82 (12.69)	24.16 (df=2,92) p<0.001	0.344			34/25/38
TSI-2: Externalization	28.91 (21.46)	38.76 (22.82)	5.79 (7.17)	18.81 (df=2,92) p<0.001	0.290			34/25/38
TSI-2: Somatization	11.73 (9.09)	10.32 (6.08)	3.79 (3.73)	14.50 (df=2,92) p<0.001	0.240			34/25/38

^η² = partial eta squared (effect size). Hedges g = standardized mean difference between cPTSD and BPD. CI = 95% confidence interval. P values are adjusted for multiple testing using the Benjamini–Hochberg false-discovery rate (within domain). Results are from ANCOVAs adjusted for age and medication status.^

### 3.3 Borderline-personality-disorder-related symptoms

As shown in **Table 3**, both groups displayed significantly higher levels of borderline-related symptom expression when compared to the healthy control group, but no significant difference between patient groups. These results were obtained from ANCOVAs adjusted for age and medication status.

**Table 3:** Group comparisons for BPD-related symptoms.

Questionnaire	Groups			ANCOVA		Significant *post-hoc* tests: cPTSD vs. BPD		
	cPTSD M (SD)	BPD M (SD)	Control M (SD)	F(df), p	η²	Hedges g	95% CI	n (cPTSD/BPD/HC)
Borderline symptom severity								
BSL-23	1.407(1.073)	1.557(0.806)	0.170(0.227)	F(2, 92) = 24.86, p <.001	.352			34/25/38

^η² = partial eta squared (effect size). Hedges g = standardized mean difference between cPTSD and BPD. CI = 95% confidence interval. Results are from ANCOVAs adjusted for age and medication status. All p values were adjusted for multiple testing using the Benjamini Hochberg false discovery rate procedure.^

### 3.4 General psychopathology

**Figure 2:**
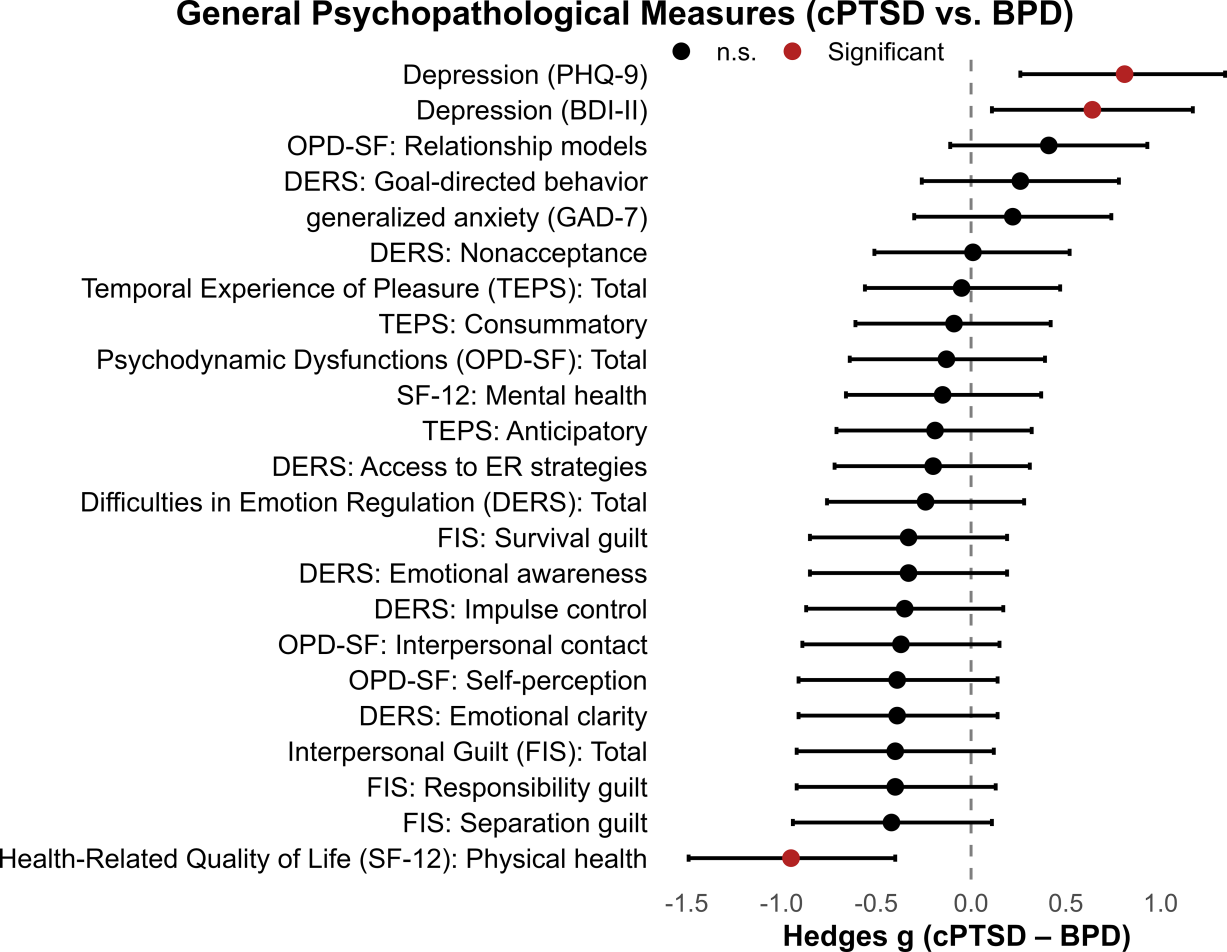
General psychopathological measures in patients with cPTSD and BPD. Forest plot of standardized mean differences (Hedges g) with 95% confidence intervals comparing cPTSD and BPD groups across general psychopathological symptom domains. Positive values indicate higher scores in the cPTSD group; negative values indicate higher scores in the BPD group.

We found significant group effects across all domains of general psychopathology (see **Table 4**). Specifically, both clinical groups reported significantly higher symptoms of depression, anxiety and difficulties in emotion regulation when compared to healthy controls (all *post-hoc* comparisons between HCs and patient groups *P* < 0.001). When comparing both patient groups, we found that patients with cPTSD showed higher scores of depression than patients with BPD (only when assessed with the PHQ-9, not for BDI-II scores). However, there were no differences in symptoms of anxiety and difficulties in emotion regulation between patient groups. Physical health perception was reduced in patients with cPTSD when compared to both healthy controls and patients with BPD, whereas there was no difference between patient groups in mental health perception. Personality structure and functioning assessed across the areas of self-perception, interpersonal contact, and relationship models, were lower in both patient groups when compared to healthy controls, but no differences between patient groups were found. Except for the “separation guilt” subscale from the FIS-scale, where only patients with BPD had higher scores when compared to healthy controls. These results were obtained from ANCOVAs adjusted for age and medication status. Multivariate analyses confirmed robust overall group effects for the DERS and OPD domains (all Wilks’ Λ < 0.50, p <.001). Standardized mean differences between cPTSD and BPD across general psychopathological measures are shown in **Figure 2**.

**Table 4:** Group comparisons for general psychopathological measures.

Questionnaire	Groups			ANCOVA		Significant *post-hoc* tests: cPTSD vs. BPD		
	cPTSD M (SD)	BPD M (SD)	Control M (SD)	F(df), p	η²	Hedges g	95% CI	n (cPTSD/BPD/HC)
Depressive symptoms								
BDI	34.50 (10.41)	27.32 (12.24)	4.44 (4.80)	76.52 (df=2,92) p<0.001	0.625	0.64	[0.11, 1.17]	34/25/38
PHQ-9	16.56 (5.19)	12.60 (4.52)	3.50 (2.56)	67.23 (df=2,92) p<0.001	0.594	0.81	[0.26, 1.34]	34/25/38
Anxiety-related symptoms								
GAD-7	11.44 (4.20)	10.48 (4.52)	1.71 (2.64)	59.28 (df=2,92) p<0.001	0.563			34/25/38
Emotion regulation								
DERS: Total score	117.77 (26.32)	123.92 (24.59)	57.95 (15.97)	58.01 (df=2,92) p<0.001	0.558			34/25/38
DERS: Nonacceptance	19.77 (5.97)	19.72 (6.93)	10.11 (3.85)	22.91 (df=2,92) p<0.001	0.332			34/25/38
DERS: Goal-directed behavior	19.35 (4.71)	18.08 (4.94)	9.03 (3.77)	37.53 (df=2,92) p<0.001	0.449			34/25/38
DERS: Impulse control	17.12 (6.88)	19.40 (5.79)	8.53 (2.83)	22.48 (df=2,92) p<0.001	0.328			34/25/38
DERS: Emotional awareness	18.91 (4.93)	20.64 (5.47)	12.08 (5.21)	17.28 (df=2,92) p<0.001	0.273			34/25/38
DERS: Access to ER strategies	27.53 (8.13)	29.08 (6.79)	11.32 (4.59)	47.73 (df=2,92) p<0.001	0.509			34/25/38
DERS: Emotional clarity	15.09 (4.95)	17.00 (4.90)	6.89 (2.57)	38.80 (df=2,92) p<0.001	0.458			34/25/38
Health-related functioning								
SF-12: Physical health perception	42.62 (13.23)	53.27 (7.57)	54.75 (5.09)	13.39 (df=2,92) p<0.001	0.225	-0.95	[-1.49, -0.40]	34/25/38
SF-12: Mental health perception	27.88 (8.80)	29.17 (9.03)	51.77 (8.35)	58.28 (df=2,92) p<0.001	0.559			34/25/38
Psychodynamic dysfunction								
OPD-SQ SF: Total score	29.79 (9.79)	30.96 (8.66)	7.97 (6.43)	60.38 (df=2,92) p<0.001	0.568			34/25/38
OPD-SQ SF: Self-perception	8.71 (4.44)	10.28 (3.52)	0.74 (1.50)	53.53 (df=2,92) p<0.001	0.538			34/25/38
OPD-SQ SF: Interpersonal contact	9.18 (3.11)	10.36 (3.28)	2.76 (2.68)	43.28 (df=2,92) p<0.001	0.485			34/25/38
OPD-SQ SF: Relationship models	11.91 (3.69)	10.32 (4.04)	4.47 (3.57)	33.22 (df=2,92) p<0.001	0.419			34/25/38
Pleasure experience								
TEPS: Total Score	64.97 (17.07)	65.76 (17.86)	85.61 (11.52)	12.42 (df=2,92) p<0.001	0.213			34/25/38
TEPS: Anticipatory pleasure	33.73 (8.04)	35.44 (9.69)	46.00 (6.90)	15.70 (df=2,92) p<0.001	0.254			34/25/38
TEPS: Consummatory pleasure	31.23 (11.02)	32.12 (7.06)	39.60 (5.50)	6.15 (df=2,92) p=0.010	0.118			34/25/38
Guilt-related distress								
FIS: Total score	50.35 (30.20)	60.40 (15.07)	32.68 (18.98)	7.76 (df=2,92) p=0.003	0.144			34/25/38
FIS: Survival guilt	18.03 (11.23)	21.28 (7.72)	8.16 (5.19)	15.35 (df=2,92) p<0.001	0.250			34/25/38
FIS: Separation guilt	11.21 (7.70)	13.96 (4.72)	9.26 (6.10)	2.35 (df=2,92) p=0.249	0.049			34/25/38
FIS: Omnipotent responsibility guilt	21.12 (12.57)	25.16 (5.45)	15.26 (9.32)	4.88 (df=2,92) p=0.030 *	0.096			34/25/38

^η² = partial eta squared (effect size). Hedges g = standardized mean difference between cPTSD and BPD. CI = 95% confidence interval. P values are adjusted for multiple testing using the Benjamini–Hochberg false-discovery rate (within domain). Results are from ANCOVAs adjusted for age and medication status.^

### 3.5 Exploratory classification

The LASSO model using trauma-related scales discriminated cPTSD from BPD with good performance. The optimal model (λ = 0.0365) achieved an out-of-fold AUC of 0.84 (95% CI [0.80, 0.89]), significantly above chance level (AUC = 0.5). Corresponding classification metrics were accuracy 0.81, balanced accuracy 0.80, sensitivity 0.87, and specificity 0.74. Across the 50 resampled cross-validation folds, AUC values ranged from 0.78 to 0.87, indicating stable discriminative performance. The strongest predictors retained at the optimal λ were adverse childhood experiences (ACE-D), PTSD symptom severity (PDS severity), and dissociation (FDS-20), with positive coefficients indicating higher values in the cPTSD group. Smaller but nonzero coefficients were observed for trauma symptom scales such as TSI intrusive experiences and functional impairment. Standardized coefficients are listed in **Supplementary Table S2**. Full standardized coefficients of the final model are reported in **Supplementary Table S2**.

## 4 Discussion

The aim of the present study was to systematically investigate the symptomatology of complex posttraumatic stress disorder and borderline personality disorder. These diagnoses were based on structured clinical interviews and ICD-11 criteria, but we did not include ICD-11 specific instruments such as the ITQ or ITI, which means that the findings should be considered preliminary regarding ICD-11 cPTSD. Furthermore, diagnoses were based on structured clinical interviews and ICD-11 criteria, but DSM-oriented instruments were employed to capture symptom expression. This ensured use of validated, widely applied measures and comparability with previous research. At the same time, it limits the extent to which our findings can be directly mapped onto ICD-11 constructs, as ICD-11-specific instruments were not applied. Using a broad range of standardized psychometric measures, we sought to identify areas of overlap and divergence between the two disorders and to empirically assess distinctions reported in earlier studies. We found that trauma-related domains were the most prominent distinction between the two clinical groups. Specifically, patients with cPTSD consistently reported higher symptom levels on measures assessing adverse childhood experiences, exposure to traumatic events, dissociative symptoms and overall PTSD symptom severity as well as impairment. Patient groups were largely similar in domains of general psychopathology, except for increased affective symptoms and lower physical health perception in patients with cPTSD. Finally, we found no differences in borderline-related symptoms.

Our observation of increased exposure to adverse childhood experiences and traumatic events supports the role of trauma as a central etiological factor for cPTSD. Given that as opposed to BPD, trauma exposure is a core diagnostic requirement for cPTSD in ICD-11, these differences may reflect the diagnostic criterion itself. However, our observation of both greater intensity of trauma-related symptoms and a higher incidence of adverse childhood experiences and traumatic events in patients with cPTSD suggests that trauma-exposure in cPTSD is not only more prevalent but also more severe. While exposure to adverse life experiences is common in BPD (16), the increased trauma-related functional impairment and symptom severity observed in patients with cPTSD suggest that trauma severity may be a key distinguishing factor between the two disorders. This pattern may also reflect a cumulative or self-reinforcing process, in which early adverse experiences increase vulnerability to further traumatic events across the lifespan, ultimately resulting in more severe trauma-related symptoms and the development of a disorder in which trauma consequences are central.

Furthermore, dissociative symptoms were significantly higher in the cPTSD group. Although dissociation is a relevant feature in both disorders (6), in cPTSD it is often associated with trauma related structural dissociation, which may be more severe than the stress related dissociation typically observed in BPD (63, 64). This finding aligns with research identifying dissociation as a core feature of complex trauma responses (65) and when cPTSD symptoms co-occur with dissociative experiences, health outcomes tend to be more severe (66), consistent with our observation of increased general symptom expression in patients with cPTSD. These findings raise the question of whether dissociative symptoms should be more explicitly included in the diagnostic criteria for cPTSD, given their apparent clinical relevance and impact on severity.

Additionally, we failed to observe differences between patient groups in BPD-related symptom severity. While this may suggest clinical similarity, this finding should be interpreted with caution. The scale employed in this study (BSL-23) primarily captures the overall severity of emotional distress and core features commonly associated with BPD, such as affective instability, self-contempt, and identity disturbance, but it does not comprehensively assess the full range of diagnostic criteria (61, 67). Specific features more characteristic of BPD, such as fear of abandonment, unstable interpersonal relationships, impulsivity, and self-injurious behavior (68–70), are only partially or indirectly represented in the BSL-23 and were not directly assessed using other instruments in the current study. This measurement strategy therefore likely reduced sensitivity to BPD-specific features, which limits the strength of our “no difference” conclusion.

Differences in depressive symptom severity were also observed. Patients with cPTSD reported significantly higher scores than patients with BPD on both the PHQ-9 and the BDI-II. This pattern strengthens the evidence for elevated depressive symptom burden in cPTSD and indicates that the effect is not dependent on the specific depression measure employed. This finding aligns with our observation of reduced physical health perception and increased trauma-related functional impairment in patients with cPTSD. At the same time, the overlap between PTSD and depression symptom domains, particularly in the PHQ-9, should be kept in mind when interpreting these results. Beyond disorder-specific profiles, the results revealed a notable degree of symptomatic convergence, particularly in the area of general psychopathology. In all assessed psychopathological domains, both clinical groups differed significantly from healthy controls but not from each other. These domains include anxiety-related symptoms, emotion regulation difficulties, physiological and mental health perception, personality structure and functioning, pleasure experience, and guilt-related distress (with separation guilt as an exception). This consistent pattern of heightened distress compared to healthy controls highlights the overall severity and burden of both disorders. It also supports findings suggesting that cPTSD and BPD share core features of psychological dysfunction (14) and may help explain the substantial comorbidity between the two diagnoses (6). Furthermore, our findings can also be understood in light of dimensional models of psychopathology, such as the Hierarchical Taxonomy of Psychopathology (HiTOP; 13, 71). HiTOP conceptualizes symptoms as clustering along broader spectra, for example internalizing or externalizing. Within this framework, the overlap we observed between cPTSD and BPD in domains such as general psychopathology and emotion dysregulation may reflect shared positions on these higher-order dimensions. By contrast, the stronger trauma-related symptom burden and dissociation in cPTSD may indicate additional transdiagnostic features that shape a distinct clinical profile. Considering the results from this dimensional perspective helps explain why comorbidity between cPTSD and BPD is common, while also clarifying how differences in symptom severity and accumulation can contribute to diagnostic divergence.

Finally, as an exploratory step, we implemented a penalized logistic regression (LASSO) model using trauma-related measures to discriminate between cPTSD and BPD. The model showed good discriminative performance, with strongest predictors being adverse childhood experiences, PTSD symptom severity, and dissociation scores. This indicates that trauma-related domains carry discriminative signal beyond overall psychopathology. These results suggest that trauma-related measures may help distinguish cPTSD from BPD. However, given the modest sample size and the exploratory nature of this approach, findings should be interpreted with caution and considered primarily hypothesis-generating for future research in larger cohorts.

### 4.1 Limitations

Several limitations of the present study need to be acknowledged. First, the relatively small sample size limited the statistical power to detect subtle group differences, and the fact that we only included female participants reduced the generalizability of our results. Both cPTSD and BPD are heterogeneous, and comorbidities may have influenced our results. Second, self-report scales are susceptible to social desirability, recall bias, and limitations in self-awareness. Although we used well-validated, widely applied instruments, the addition of clinician- and observer-based assessments could strengthen future studies by providing a more nuanced understanding of symptomatology. Third, since we used only one measure for BPD symptoms (the BSL-23), the full range of BPD criteria was not comprehensively assessed. While the BSL-23 is a well-validated measure of global borderline distress, it does not fully capture features such as fear of abandonment, unstable relationships, impulsivity, and self-harm. This limitation reduces sensitivity to BPD-specific features and constrains the interpretation of our finding of no difference between cPTSD and BPD in BSL-23 scores. Future studies should incorporate complementary clinician-rated or self-report measures to better capture the multidimensional nature of BPD. Fourth, an effect of medication cannot be ruled out, as 12 patients with cPTSD and 13 patients with BPD were receiving antidepressant medication. Fifth, patients with BPD were significantly younger than patients with cPTSD. While this represents a limitation of the study and was influenced by recruitment difficulties and the characteristics of the patient sample, it may also support the proposed theory that cPTSD and BPD exist on a trauma-related severity continuum. In this view, early trauma-driven BPD presentations may shift toward more complex dissociative cPTSD profiles later in adulthood, particularly following prolonged interpersonal trauma (6, 72). Accordingly, age-related differences may reflect not only chronological factors but also the cumulative effects of trauma on symptom development. Sixth, although participants with active substance use disorder were excluded, the absence of a systematic lifetime assessment represents a limitation, since previous or remitted substance use may still have influenced the outcomes. Seventh, although all patients and healthy controls underwent a standardized diagnostic assessment with the SCID, we did not administer ICD 11 specific diagnostic instruments such as the International Trauma Questionnaire (ITQ; 73) or International Trauma Interview (ITI; 74). This represents a limitation, since the SCID is primarily DSM oriented and does not fully capture the specific construct of ICD 11 cPTSD. Therefore, while patients were included only if they met ICD 11 criteria for cPTSD or BPD, the absence of ITQ or ITI data limits the strength of claims about ICD 11 cPTSD. More broadly, our reliance on DSM-based instruments for symptom assessment means that ICD-11 constructs were operationalized using DSM-oriented tools. While this approach ensured use of psychometrically robust and widely applied measures, it constrains the construct validity of our conclusions within the ICD-11 framework and should be regarded as a central limitation. Eighth, the modest sample size also limited the robustness of multivariate models and exploratory classification analyses. These analyses provide important complementary insights but should be replicated in larger samples to confirm stability of the findings. Ninth, all participants were women, and only sex assigned at birth was recorded, while gender identity was not systematically assessed. This strategy limits the generalizability of our findings to men and gender-diverse populations. Future research should examine whether the observed patterns extend across sexes and genders. Finally, the cross-sectional design limits causal inferences, replication with larger samples and longitudinal designs is warranted.

## 5 Conclusion

Taken together, while both disorders showed comparable levels of general psychopathology and BPD-related symptoms, patients with cPTSD exhibited a stronger trauma history, higher levels of dissociation, and more pronounced posttraumatic stress symptoms, pointing to a distinct trauma-related profile. Accordingly, these characteristics go beyond general psychological distress and represent specific distinguishing features, consistent with prior research using latent profile analysis, exploratory structural equation modeling, or confirmatory factor analysis analysis (e.g. 3, 7–10). The current findings therefore add empirical support to the argument that cPTSD and BPD should remain diagnostically distinct categories rather than being conceptualized as overlapping variants of a single disorder. In addition, the multivariate and exploratory classification analyses conducted in this study provide complementary evidence that trauma-related symptom domains meaningfully distinguish cPTSD from BPD. These results should be interpreted with caution due to the modest sample size but highlight promising directions for future research. However, since ICD-11 specific instruments such as the ITQ or ITI were not applied, the conclusions about ICD-11 cPTSD should be regarded as tentative. The observed differences in symptom expression are not only diagnostically relevant but also carry important therapeutic consequences. The stronger expression of trauma- and dissociation-related symptoms in cPTSD indicates the need for targeted trauma-focused interventions, such as Eye Movement Desensitization and Reprocessing (EMDR; 75), often preceded by stabilization-focused approaches such as Skills Training in Affective and Interpersonal Regulation (STAIR; 76). In contrast, conventional BPD treatments like dialectical behavior therapy (DBT) or Mentalization-Based Treatment (MBT; 77) may be particularly effective when focused on emotion regulation, behavioral stabilization, and reduction of self-harming behaviors (78). More broadly, sequencing care so that stabilization precedes trauma-focused work is emphasized in international guidelines (79) and is especially relevant given the severity of trauma-related impairment observed in the cPTSD group. Failure to adequately screen for trauma and provide suitable treatment can lead to long-term frustration for patients, potentially resulting in recurrent hospitalizations, substance abuse, and lower functional levels (80).

## Data Availability

The raw data supporting the conclusions of this article and corresponding R analysis scripts can be found at: https://osf.io/7268a.
