# Supplementary material for: Beyond diagnosis: symptom patterns across complex PTSD and borderline personality disorder
Source: Front Psychiatry. 2025 Oct 30;16:1668821. doi: 10.3389/fpsyt.2025.1668821 (PMC12612632; doi:10.3389/fpsyt.2025.1668821)
Supplement: Supplementary file 1 [file DataSheet1.docx]

Supplementary Material

**Beyond Diagnosis: Symptom Patterns Across Complex PTSD and Borderline Personality Disorder**

Joe J. Simon^12^, Kristin Spiegler^3^, Kadiatou Coulibaly^1^; Marion A. Stopyra^14^; Hans-Christoph Friederich^12^; Oliver Gruber^5^; Christoph Nikendei^1^.

# Supplementary Data

Supplementary Material should be uploaded separately on submission. Please include any supplementary data, figures and/or tables.

Supplementary material is not typeset so please ensure that all information is clearly presented, the appropriate caption is included in the file and not in the manuscript, and that the style conforms to the rest of the article.

# Supplementary Figures and Tables

**Table S1. Summary Table Participant Medication**

| **Group** | **Medication Class** | **Total Count** | **Specific agents (with counts)** |
| --- | --- | --- | --- |
| BPD | Selective Serotonin Reuptake Inhibitors | 13 | Citalopram (N=4), Escitalopram (N=3), Fluoxetin (N=3), Sertraline (N=4) |
| BPD | Tricyclic Antidepressant | 1 | Amitriptyline (N=1) |
| cPTSD | Selective Serotonin Reuptake Inhibitors | 16 | Citalopram (N=4), Escitalopram (N=5), Fluoxetin (N=3), Sertraline (N=4) |

**Table S2. Missingness by group (% per variable)**

| Group | Variable | n_missing | perc_missing |
| --- | --- | --- | --- |
| Control | Age | 0 | 0 |
| Control | Educational attainment | 0 | 0 |
| Control | Vocational qualification | 0 | 0 |
| Control | Premorbid intelligence (MWT-B) | 0 | 0 |
| Control | skPTBS: Total score | 0 | 0 |
| Control | ACE-D | 0 | 0 |
| Control | LEC-5 | 9 | 23.684 |
| Control | PDS: Severity | 0 | 0 |
| Control | PDS: Functional impairment | 0 | 0 |
| Control | FDS-20 | 0 | 0 |
| Control | TSI-2: Anxious arousal | 0 | 0 |
| Control | TSI-2: Depression | 0 | 0 |
| Control | TSI-2: Anger/irritability | 0 | 0 |
| Control | TSI-2: Intrusive experiences | 0 | 0 |
| Control | TSI-2: DepressionA | 0 | 0 |
| Control | TSI-2: DepressionIS | 0 | 0 |
| Control | TSI-2: Somatic preoccupation | 0 | 0 |
| Control | TSI-2: Sexual disturbance | 0 | 0 |
| Control | TSI-2: Suicidality | 0 | 0 |
| Control | TSI-2: Insecure attachment | 0 | 0 |
| Control | TSI-2: Impaired self-reference | 0 | 0 |
| Control | TSI-2: Tension reduction behavior | 0 | 0 |
| Control | TSI-2: Self-disturbance | 0 | 0 |
| Control | TSI-2: Trauma-specific dysregulation | 0 | 0 |
| Control | TSI-2: Externalization | 0 | 0 |
| Control | TSI-2: Somatization | 0 | 0 |
| Control | BSL-23 | 0 | 0 |
| Control | BDI | 0 | 0 |
| Control | PHQ-9 | 0 | 0 |
| Control | GAD-7 | 0 | 0 |
| Control | DERS: Total score | 0 | 0 |
| Control | DERS: Nonacceptance | 0 | 0 |
| Control | DERS: Goal-directed behavior | 0 | 0 |
| Control | DERS: Impulse control | 0 | 0 |
| Control | DERS: Emotional awareness | 0 | 0 |
| Control | DERS: Access to ER strategies | 0 | 0 |
| Control | DERS: Emotional clarity | 0 | 0 |
| Control | SF-12: Physical health perception | 0 | 0 |
| Control | SF-12: Mental health perception | 0 | 0 |
| Control | OPD-SQ SF: Total score | 0 | 0 |
| Control | OPD-SQ SF: Self-perception | 0 | 0 |
| Control | OPD-SQ SF: Interpersonal contact | 0 | 0 |
| Control | OPD-SQ SF: Relationship models | 0 | 0 |
| Control | TEPS: Total score | 0 | 0 |
| Control | TEPS: Anticipatory pleasure | 0 | 0 |
| Control | TEPS: Consummatory pleasure | 0 | 0 |
| Control | FIS: Total score | 0 | 0 |
| Control | FIS: Survival guilt | 0 | 0 |
| Control | FIS: Separation guilt | 0 | 0 |
| Control | FIS: Omnipotent responsibility guilt | 0 | 0 |
| cPTSD | Age | 0 | 0 |
| cPTSD | Educational attainment | 0 | 0 |
| cPTSD | Vocational qualification | 0 | 0 |
| cPTSD | Premorbid intelligence (MWT-B) | 2 | 5.882 |
| cPTSD | skPTBS: Total score | 3 | 8.823529412 |
| cPTSD | ACE-D | 0 | 0 |
| cPTSD | LEC-5 | 2 | 5.882 |
| cPTSD | PDS: Severity | 0 | 0 |
| cPTSD | PDS: Functional impairment | 0 | 0 |
| cPTSD | FDS-20 | 1 | 2.941 |
| cPTSD | TSI-2: Anxious arousal | 0 | 0 |
| cPTSD | TSI-2: Depression | 0 | 0 |
| cPTSD | TSI-2: Anger/irritability | 0 | 0 |
| cPTSD | TSI-2: Intrusive experiences | 0 | 0 |
| cPTSD | TSI-2: DepressionA | 0 | 0 |
| cPTSD | TSI-2: DepressionIS | 0 | 0 |
| cPTSD | TSI-2: Somatic preoccupation | 0 | 0 |
| cPTSD | TSI-2: Sexual disturbance | 0 | 0 |
| cPTSD | TSI-2: Suicidality | 0 | 0 |
| cPTSD | TSI-2: Insecure attachment | 0 | 0 |
| cPTSD | TSI-2: Impaired self-reference | 0 | 0 |
| cPTSD | TSI-2: Tension reduction behavior | 0 | 0 |
| cPTSD | TSI-2: Self-disturbance | 0 | 0 |
| cPTSD | TSI-2: Trauma-specific dysregulation | 0 | 0 |
| cPTSD | TSI-2: Externalization | 0 | 0 |
| cPTSD | TSI-2: Somatization | 0 | 0 |
| cPTSD | BSL-23 | 0 | 0 |
| cPTSD | BDI | 0 | 0 |
| cPTSD | PHQ-9 | 0 | 0 |
| cPTSD | GAD-7 | 0 | 0 |
| cPTSD | DERS: Total score | 0 | 0 |
| cPTSD | DERS: Nonacceptance | 0 | 0 |
| cPTSD | DERS: Goal-directed behavior | 0 | 0 |
| cPTSD | DERS: Impulse control | 0 | 0 |
| cPTSD | DERS: Emotional awareness | 0 | 0 |
| cPTSD | DERS: Access to ER strategies | 0 | 0 |
| cPTSD | DERS: Emotional clarity | 0 | 0 |
| cPTSD | SF-12: Physical health perception | 0 | 0 |
| cPTSD | SF-12: Mental health perception | 0 | 0 |
| cPTSD | OPD-SQ SF: Total score | 0 | 0 |
| cPTSD | OPD-SQ SF: Self-perception | 0 | 0 |
| cPTSD | OPD-SQ SF: Interpersonal contact | 0 | 0 |
| cPTSD | OPD-SQ SF: Relationship models | 0 | 0 |
| cPTSD | TEPS: Total score | 0 | 0 |
| cPTSD | TEPS: Anticipatory pleasure | 0 | 0 |
| cPTSD | TEPS: Consummatory pleasure | 0 | 0 |
| cPTSD | FIS: Total score | 0 | 0 |
| cPTSD | FIS: Survival guilt | 0 | 0 |
| cPTSD | FIS: Separation guilt | 0 | 0 |
| cPTSD | FIS: Omnipotent responsibility guilt | 0 | 0 |
| BPD | Age | 0 | 0 |
| BPD | Educational attainment | 0 | 0 |
| BPD | Vocational qualification | 0 | 0 |
| BPD | Premorbid intelligence (MWT-B) | 0 | 0 |
| BPD | skPTBS: Total score | 0 | 0 |
| BPD | ACE-D | 0 | 0 |
| BPD | LEC-5 | 1 | 4 |
| BPD | PDS: Severity | 0 | 0 |
| BPD | PDS: Functional impairment | 0 | 0 |
| BPD | FDS-20 | 0 | 0 |
| BPD | TSI-2: Anxious arousal | 0 | 0 |
| BPD | TSI-2: Depression | 0 | 0 |
| BPD | TSI-2: Anger/irritability | 0 | 0 |
| BPD | TSI-2: Intrusive experiences | 0 | 0 |
| BPD | TSI-2: DepressionA | 0 | 0 |
| BPD | TSI-2: DepressionIS | 0 | 0 |
| BPD | TSI-2: Somatic preoccupation | 0 | 0 |
| BPD | TSI-2: Sexual disturbance | 0 | 0 |
| BPD | TSI-2: Suicidality | 0 | 0 |
| BPD | TSI-2: Insecure attachment | 0 | 0 |
| BPD | TSI-2: Impaired self-reference | 0 | 0 |
| BPD | TSI-2: Tension reduction behavior | 0 | 0 |
| BPD | TSI-2: Self-disturbance | 0 | 0 |
| BPD | TSI-2: Trauma-specific dysregulation | 0 | 0 |
| BPD | TSI-2: Externalization | 0 | 0 |
| BPD | TSI-2: Somatization | 0 | 0 |
| BPD | BSL-23 | 0 | 0 |
| BPD | BDI | 0 | 0 |
| BPD | PHQ-9 | 0 | 0 |
| BPD | GAD-7 | 0 | 0 |
| BPD | DERS: Total score | 0 | 0 |
| BPD | DERS: Nonacceptance | 0 | 0 |
| BPD | DERS: Goal-directed behavior | 0 | 0 |
| BPD | DERS: Impulse control | 0 | 0 |
| BPD | DERS: Emotional awareness | 0 | 0 |
| BPD | DERS: Access to ER strategies | 0 | 0 |
| BPD | DERS: Emotional clarity | 0 | 0 |
| BPD | SF-12: Physical health perception | 0 | 0 |
| BPD | SF-12: Mental health perception | 0 | 0 |
| BPD | OPD-SQ SF: Total score | 0 | 0 |
| BPD | OPD-SQ SF: Self-perception | 0 | 0 |
| BPD | OPD-SQ SF: Interpersonal contact | 0 | 0 |
| BPD | OPD-SQ SF: Relationship models | 0 | 0 |
| BPD | TEPS: Total score | 0 | 0 |
| BPD | TEPS: Anticipatory pleasure | 0 | 0 |
| BPD | TEPS: Consummatory pleasure | 0 | 0 |
| BPD | FIS: Total score | 0 | 0 |
| BPD | FIS: Survival guilt | 0 | 0 |
| BPD | FIS: Separation guilt | 0 | 0 |
| BPD | FIS: Omnipotent responsibility guilt | 0 | 0 |

Note: Missingness per group was generally low (≤ 5% per variable across groups).

**Table S3. Shapiro–Wilk normality tests by group**

| Group | Variable | statistic | p |
| --- | --- | --- | --- |
| Control | Age | 0.953 | 0.116 |
| cPTSD | Age | 0.922 | 0.019 |
| BPD | Age | 0.910 | 0.030 |
| Control | Educational attainment | 0.558 | 0.000 |
| cPTSD | Educational attainment | 0.746 | 0.000 |
| BPD | Educational attainment | 0.755 | 0.000 |
| Control | Vocational qualification | 0.827 | 0.000 |
| cPTSD | Vocational qualification | 0.864 | 0.001 |
| BPD | Vocational qualification | 0.783 | 0.000 |
| Control | Premorbid intelligence (MWT-B) | 0.943 | 0.052 |
| cPTSD | Premorbid intelligence (MWT-B) | 0.918 | 0.018 |
| BPD | Premorbid intelligence (MWT-B) | 0.964 | 0.501 |
| Control | skPTBS: Total score | 0.755 | 0.000 |
| cPTSD | skPTBS: Total score | 0.894 | 0.005 |
| BPD | skPTBS: Total score | 0.971 | 0.681 |
| Control | ACE-D | 0.729 | 0.000 |
| cPTSD | ACE-D | 0.920 | 0.016 |
| BPD | ACE-D | 0.958 | 0.374 |
| Control | LEC-5 | 0.611 | 0.000 |
| cPTSD | LEC-5 | 0.936 | 0.059 |
| BPD | LEC-5 | 0.952 | 0.293 |
| Control | PDS: Severity | 0.550 | 0.000 |
| cPTSD | PDS: Severity | 0.921 | 0.020 |
| BPD | PDS: Severity | 0.921 | 0.091 |
| Control | PDS: Functional impairment | 0.391 | 0.000 |
| cPTSD | PDS: Functional impairment | 0.912 | 0.011 |
| BPD | PDS: Functional impairment | 0.878 | 0.014 |
| Control | FDS-20 | 0.771 | 0.000 |
| cPTSD | FDS-20 | 0.934 | 0.046 |
| BPD | FDS-20 | 0.932 | 0.097 |
| Control | TSI-2: Anxious arousal | 0.897 | 0.002 |
| cPTSD | TSI-2: Anxious arousal | 0.871 | 0.001 |
| BPD | TSI-2: Anxious arousal | 0.953 | 0.299 |
| Control | TSI-2: Depression | 0.738 | 0.000 |
| cPTSD | TSI-2: Depression | 0.865 | 0.001 |
| BPD | TSI-2: Depression | 0.922 | 0.058 |
| Control | TSI-2: Anger/irritability | 0.875 | 0.001 |
| cPTSD | TSI-2: Anger/irritability | 0.909 | 0.008 |
| BPD | TSI-2: Anger/irritability | 0.958 | 0.368 |
| Control | TSI-2: Intrusive experiences | 0.682 | 0.000 |
| cPTSD | TSI-2: Intrusive experiences | 0.868 | 0.001 |
| BPD | TSI-2: Intrusive experiences | 0.931 | 0.091 |
| Control | TSI-2: DepressionA | 0.668 | 0.000 |
| cPTSD | TSI-2: DepressionA | 0.878 | 0.001 |
| BPD | TSI-2: DepressionA | 0.963 | 0.482 |
| Control | TSI-2: DepressionIS | 0.621 | 0.000 |
| cPTSD | TSI-2: DepressionIS | 0.673 | 0.000 |
| BPD | TSI-2: DepressionIS | 0.933 | 0.101 |
| Control | TSI-2: Somatic preoccupation | 0.880 | 0.001 |
| cPTSD | TSI-2: Somatic preoccupation | 0.896 | 0.004 |
| BPD | TSI-2: Somatic preoccupation | 0.968 | 0.589 |
| Control | TSI-2: Sexual disturbance | 0.443 | 0.000 |
| cPTSD | TSI-2: Sexual disturbance | 0.869 | 0.001 |
| BPD | TSI-2: Sexual disturbance | 0.800 | 0.000 |
| Control | TSI-2: Suicidality | 0.313 | 0.000 |
| cPTSD | TSI-2: Suicidality | 0.813 | 0.000 |
| BPD | TSI-2: Suicidality | 0.862 | 0.003 |
| Control | TSI-2: Insecure attachment | 0.796 | 0.000 |
| cPTSD | TSI-2: Insecure attachment | 0.894 | 0.003 |
| BPD | TSI-2: Insecure attachment | 0.958 | 0.380 |
| Control | TSI-2: Impaired self-reference | 0.464 | 0.000 |
| cPTSD | TSI-2: Impaired self-reference | 0.899 | 0.004 |
| BPD | TSI-2: Impaired self-reference | 0.963 | 0.469 |
| Control | TSI-2: Tension reduction behavior | 0.565 | 0.000 |
| cPTSD | TSI-2: Tension reduction behavior | 0.888 | 0.002 |
| BPD | TSI-2: Tension reduction behavior | 0.922 | 0.056 |
| Control | TSI-2: Self-disturbance | 0.713 | 0.000 |
| cPTSD | TSI-2: Self-disturbance | 0.890 | 0.002 |
| BPD | TSI-2: Self-disturbance | 0.966 | 0.543 |
| Control | TSI-2: Trauma-specific dysregulation | 0.838 | 0.000 |
| cPTSD | TSI-2: Trauma-specific dysregulation | 0.895 | 0.003 |
| BPD | TSI-2: Trauma-specific dysregulation | 0.967 | 0.569 |
| Control | TSI-2: Externalization | 0.728 | 0.000 |
| cPTSD | TSI-2: Externalization | 0.908 | 0.007 |
| BPD | TSI-2: Externalization | 0.947 | 0.215 |
| Control | TSI-2: Somatization | 0.880 | 0.001 |
| cPTSD | TSI-2: Somatization | 0.896 | 0.004 |
| BPD | TSI-2: Somatization | 0.968 | 0.589 |
| Control | BSL-23 | 0.760 | 0.000 |
| cPTSD | BSL-23 | 0.928 | 0.027 |
| BPD | BSL-23 | 0.923 | 0.060 |
| Control | BDI | 0.850 | 0.000 |
| cPTSD | BDI | 0.965 | 0.341 |
| BPD | BDI | 0.971 | 0.677 |
| Control | PHQ-9 | 0.904 | 0.003 |
| cPTSD | PHQ-9 | 0.960 | 0.248 |
| BPD | PHQ-9 | 0.960 | 0.405 |
| Control | GAD-7 | 0.696 | 0.000 |
| cPTSD | GAD-7 | 0.950 | 0.121 |
| BPD | GAD-7 | 0.956 | 0.343 |
| Control | DERS: Total score | 0.857 | 0.000 |
| cPTSD | DERS: Total score | 0.961 | 0.255 |
| BPD | DERS: Total score | 0.986 | 0.975 |
| Control | DERS: Nonacceptance | 0.812 | 0.000 |
| cPTSD | DERS: Nonacceptance | 0.952 | 0.144 |
| BPD | DERS: Nonacceptance | 0.947 | 0.216 |
| Control | DERS: Goal-directed behavior | 0.859 | 0.000 |
| cPTSD | DERS: Goal-directed behavior | 0.905 | 0.006 |
| BPD | DERS: Goal-directed behavior | 0.939 | 0.140 |
| Control | DERS: Impulse control | 0.834 | 0.000 |
| cPTSD | DERS: Impulse control | 0.963 | 0.288 |
| BPD | DERS: Impulse control | 0.964 | 0.490 |
| Control | DERS: Emotional awareness | 0.861 | 0.000 |
| cPTSD | DERS: Emotional awareness | 0.948 | 0.108 |
| BPD | DERS: Emotional awareness | 0.957 | 0.361 |
| Control | DERS: Access to ER strategies | 0.655 | 0.000 |
| cPTSD | DERS: Access to ER strategies | 0.940 | 0.062 |
| BPD | DERS: Access to ER strategies | 0.938 | 0.131 |
| Control | DERS: Emotional clarity | 0.752 | 0.000 |
| cPTSD | DERS: Emotional clarity | 0.974 | 0.584 |
| BPD | DERS: Emotional clarity | 0.963 | 0.470 |
| Control | SF-12: Physical health perception | 0.812 | 0.000 |
| cPTSD | SF-12: Physical health perception | 0.961 | 0.257 |
| BPD | SF-12: Physical health perception | 0.949 | 0.234 |
| Control | SF-12: Mental health perception | 0.804 | 0.000 |
| cPTSD | SF-12: Mental health perception | 0.908 | 0.008 |
| BPD | SF-12: Mental health perception | 0.959 | 0.393 |
| Control | OPD-SQ SF: Total score | 0.893 | 0.002 |
| cPTSD | OPD-SQ SF: Total score | 0.948 | 0.111 |
| BPD | OPD-SQ SF: Total score | 0.916 | 0.043 |
| Control | OPD-SQ SF: Self-perception | 0.571 | 0.000 |
| cPTSD | OPD-SQ SF: Self-perception | 0.964 | 0.318 |
| BPD | OPD-SQ SF: Self-perception | 0.941 | 0.156 |
| Control | OPD-SQ SF: Interpersonal contact | 0.849 | 0.000 |
| cPTSD | OPD-SQ SF: Interpersonal contact | 0.947 | 0.102 |
| BPD | OPD-SQ SF: Interpersonal contact | 0.981 | 0.903 |
| Control | OPD-SQ SF: Relationship models | 0.934 | 0.027 |
| cPTSD | OPD-SQ SF: Relationship models | 0.918 | 0.014 |
| BPD | OPD-SQ SF: Relationship models | 0.950 | 0.247 |
| Control | TEPS: Total score | 0.876 | 0.001 |
| cPTSD | TEPS: Total score | 0.923 | 0.020 |
| BPD | TEPS: Total score | 0.985 | 0.967 |
| Control | TEPS: Anticipatory pleasure | 0.934 | 0.027 |
| cPTSD | TEPS: Anticipatory pleasure | 0.914 | 0.011 |
| BPD | TEPS: Anticipatory pleasure | 0.956 | 0.335 |
| Control | TEPS: Consummatory pleasure | 0.838 | 0.000 |
| cPTSD | TEPS: Consummatory pleasure | 0.941 | 0.067 |
| BPD | TEPS: Consummatory pleasure | 0.976 | 0.791 |
| Control | FIS: Total score | 0.858 | 0.000 |
| cPTSD | FIS: Total score | 0.797 | 0.000 |
| BPD | FIS: Total score | 0.989 | 0.994 |
| Control | FIS: Survival guilt | 0.905 | 0.003 |
| cPTSD | FIS: Survival guilt | 0.852 | 0.000 |
| BPD | FIS: Survival guilt | 0.963 | 0.484 |
| Control | FIS: Separation guilt | 0.909 | 0.005 |
| cPTSD | FIS: Separation guilt | 0.925 | 0.022 |
| BPD | FIS: Separation guilt | 0.933 | 0.104 |
| Control | FIS: Omnipotent responsibility guilt | 0.896 | 0.002 |
| cPTSD | FIS: Omnipotent responsibility guilt | 0.761 | 0.000 |
| BPD | FIS: Omnipotent responsibility guilt | 0.956 | 0.340 |

Note: p < .05 indicates deviation from normality within that group.

**Table S4. Levene’s test for equality of variances**

| Variable | Levene_F | Levene_p |
| --- | --- | --- |
| Age | 2.579 | 0.081 |
| Educational attainment | 2.755 | 0.069 |
| Vocational qualification | 0.157 | 0.855 |
| Premorbid intelligence (MWT-B) | 3.025 | 0.053 |
| skPTBS: Total score | 5.827 | 0.004 |
| ACE-D | 7.520 | 0.001 |
| LEC-5 | 7.806 | 0.001 |
| PDS: Severity | 11.042 | 0.000 |
| PDS: Functional impairment | 19.147 | 0.000 |
| FDS-20 | 18.265 | 0.000 |
| TSI-2: Anxious arousal | 7.959 | 0.001 |
| TSI-2: Depression | 11.317 | 0.000 |
| TSI-2: Anger/irritability | 11.982 | 0.000 |
| TSI-2: Intrusive experiences | 15.856 | 0.000 |
| TSI-2: DepressionA | 14.953 | 0.000 |
| TSI-2: DepressionIS | 8.249 | 0.001 |
| TSI-2: Somatic preoccupation | 17.429 | 0.000 |
| TSI-2: Sexual disturbance | 17.251 | 0.000 |
| TSI-2: Suicidality | 15.041 | 0.000 |
| TSI-2: Insecure attachment | 11.485 | 0.000 |
| TSI-2: Impaired self-reference | 14.629 | 0.000 |
| TSI-2: Tension reduction behavior | 27.779 | 0.000 |
| TSI-2: Self-disturbance | 12.615 | 0.000 |
| TSI-2: Trauma-specific dysregulation | 12.223 | 0.000 |
| TSI-2: Externalization | 23.588 | 0.000 |
| TSI-2: Somatization | 17.429 | 0.000 |
| BSL-23 | 25.828 | 0.000 |
| BDI | 9.930 | 0.000 |
| PHQ-9 | 8.703 | 0.000 |
| GAD-7 | 8.325 | 0.000 |
| DERS: Total score | 3.238 | 0.044 |
| DERS: Nonacceptance | 6.797 | 0.002 |
| DERS: Goal-directed behavior | 2.303 | 0.106 |
| DERS: Impulse control | 11.376 | 0.000 |
| DERS: Emotional awareness | 0.276 | 0.759 |
| DERS: Access to ER strategies | 7.912 | 0.001 |
| DERS: Emotional clarity | 9.240 | 0.000 |
| SF-12: Physical health perception | 17.365 | 0.000 |
| SF-12: Mental health perception | 0.230 | 0.795 |
| OPD-SQ SF: Total score | 2.711 | 0.072 |
| OPD-SQ SF: Self-perception | 16.313 | 0.000 |
| OPD-SQ SF: Interpersonal contact | 1.209 | 0.303 |
| OPD-SQ SF: Relationship models | 0.159 | 0.854 |
| TEPS: Total score | 3.030 | 0.053 |
| TEPS: Anticipatory pleasure | 0.920 | 0.402 |
| TEPS: Consummatory pleasure | 7.798 | 0.001 |
| FIS: Total score | 3.314 | 0.041 |
| FIS: Survival guilt | 6.237 | 0.003 |
| FIS: Separation guilt | 3.421 | 0.037 |
| FIS: Omnipotent responsibility guilt | 2.855 | 0.063 |

Note: p < .05 indicates heteroscedasticity; Games–Howell post hoc tests were applied when appropriate.

**Table S5. Standardized coefficients from the LASSO model discriminating cPTSD vs BPD**

| **Predictor** | **Standardized coefficient (β)** |
| --- | --- |
| TSI Anger/Irritability (TSI_ANG) | 0.93 |
| PTSD Symptom Severity (PDS_Severity) | -0.86 |
| Screening for cPTSD (skPTSD_total_value) | -0.47 |
| Adverse Childhood Experiences (ACE-D) | -0.34 |
| TSI Sexual Concerns (TSI_SXD) | 0.13 |
| TSI Intrusive Experiences (TSI_IE) | -0.10 |
| TSI Dissociation (TSI_DIS) | 0.09 |
| TSI Somatization (TSI_SOM) | 0.06 |
| TSI Defensive Avoidance (TSI_DA) | -0.05 |
| (all other predictors) | Coefficients shrunk to 0 |

Note. Coefficients are standardized estimates at the optimal λ. Positive values indicate higher scores in the cPTSD group relative to BPD; negative values indicate higher scores in the BPD group.

**Table S6: Ranges by Group**

| **Group** | **Variable** | **max** | **min** |
| --- | --- | --- | --- |
| CON | ACE_D | 7.0 | 0.0 |
| CON | BDI | 18.0 | 0.0 |
| CON | BSL_23 | 0.782608695652174 | 0.0 |
| CON | DERS_Emo_Aware | 28.0 | 6.0 |
| CON | DERS_Emo_clarity | 16.0 | 5.0 |
| CON | DERS_Goal_dir_Beh | 23.0 | 5.0 |
| CON | DERS_Impulse_Control | 16.0 | 6.0 |
| CON | DERS_Limit_Access_ER | 30.0 | 8.0 |
| CON | DERS_Nonacceptance | 23.0 | 6.0 |
| CON | DERS_total | 103.0 | 39.0 |
| CON | FDS_20 | 11.0 | 0.0 |
| CON | FIS_Responsibility_Guilt | 30.0 | 0.0 |
| CON | FIS_Separation_Guilt | 27.0 | 0.0 |
| CON | FIS_Survival_Guilt | 17.0 | 0.0 |
| CON | FIS_total | 65.0 | 0.0 |
| CON | GAD_7 | 12.0 | 0.0 |
| CON | LEC_5 | 48.0 | 0.0 |
| CON | OPD_Interpersonal_Contact | 10.0 | 0.0 |
| CON | OPD_Relationship_Models | 13.0 | 0.0 |
| CON | OPD_SF_Total | 24.0 | 0.0 |
| CON | OPD_Self_Perception | 7.0 | 0.0 |
| CON | PDS_Funct_Impair | 5.0 | 0.0 |
| CON | PDS_Severity | 22.0 | 0.0 |
| CON | PHQ_D | 9.0 | 0.0 |
| CON | SF12_Mental_Summary | 59.78 | 28.34 |
| CON | SF12_Physical_Summary | 62.48 | 35.63 |
| CON | TEPS_ANT | 57.0 | 22.0 |
| CON | TEPS_CON | 47.0 | 17.0 |
| CON | TEPS_Total | 103.0 | 39.0 |
| CON | TSI_AA | 18.0 | 0.0 |
| CON | TSI_ANG | 15.0 | 0.0 |
| CON | TSI_D | 17.0 | 0.0 |
| CON | TSI_DA | 20.0 | 0.0 |
| CON | TSI_DIS | 9.0 | 0.0 |
| CON | TSI_F_Ext | 35.0 | 0.0 |
| CON | TSI_F_Self | 53.0 | 0.0 |
| CON | TSI_F_Soma | 14.0 | 0.0 |
| CON | TSI_F_Trauma | 54.0 | 0.0 |
| CON | TSI_IA | 18.0 | 0.0 |
| CON | TSI_IE | 19.0 | 0.0 |
| CON | TSI_ISR | 20.0 | 0.0 |
| CON | TSI_SOM | 14.0 | 0.0 |
| CON | TSI_SUI | 7.0 | 0.0 |
| CON | TSI_SXD | 11.0 | 0.0 |
| CON | TSI_TRB | 8.0 | 0.0 |
| CON | skPTSD_total_value | 110.0 | 0.0 |
| cPTSD | ACE_D | 10.0 | 0.0 |
| cPTSD | BDI | 57.0 | 14.0 |
| cPTSD | BSL_23 | 3.478260869565217 | 0.0 |
| cPTSD | DERS_Emo_Aware | 27.0 | 10.0 |
| cPTSD | DERS_Emo_clarity | 25.0 | 5.0 |
| cPTSD | DERS_Goal_dir_Beh | 25.0 | 8.0 |
| cPTSD | DERS_Impulse_Control | 30.0 | 6.0 |
| cPTSD | DERS_Limit_Access_ER | 38.0 | 8.0 |
| cPTSD | DERS_Nonacceptance | 30.0 | 6.0 |
| cPTSD | DERS_total | 160.0 | 43.0 |
| cPTSD | FDS_20 | 70.0 | 0.0 |
| cPTSD | FIS_Responsibility_Guilt | 34.0 | 0.0 |
| cPTSD | FIS_Separation_Guilt | 27.0 | 0.0 |
| cPTSD | FIS_Survival_Guilt | 33.0 | 0.0 |
| cPTSD | FIS_total | 89.0 | 0.0 |
| cPTSD | GAD_7 | 19.0 | 4.0 |
| cPTSD | LEC_5 | 110.0 | 16.0 |
| cPTSD | OPD_Interpersonal_Contact | 15.0 | 0.0 |
| cPTSD | OPD_Relationship_Models | 17.0 | 0.0 |
| cPTSD | OPD_SF_Total | 45.0 | 0.0 |
| cPTSD | OPD_Self_Perception | 16.0 | 0.0 |
| cPTSD | PDS_Funct_Impair | 9.0 | 0.0 |
| cPTSD | PDS_Severity | 47.0 | 0.0 |
| cPTSD | PHQ_D | 24.0 | 4.0 |
| cPTSD | SF12_Mental_Summary | 55.15 | 15.2 |
| cPTSD | SF12_Physical_Summary | 61.3 | 10.78 |
| cPTSD | TEPS_ANT | 49.0 | 7.0 |
| cPTSD | TEPS_CON | 47.0 | 0.0 |
| cPTSD | TEPS_Total | 91.0 | 7.0 |
| cPTSD | TSI_AA | 29.0 | 0.0 |
| cPTSD | TSI_ANG | 25.0 | 0.0 |
| cPTSD | TSI_D | 30.0 | 0.0 |
| cPTSD | TSI_DA | 30.0 | 0.0 |
| cPTSD | TSI_DIS | 71.0 | 0.0 |
| cPTSD | TSI_F_Ext | 65.0 | 0.0 |
| cPTSD | TSI_F_Self | 80.0 | 0.0 |
| cPTSD | TSI_F_Soma | 27.0 | 0.0 |
| cPTSD | TSI_F_Trauma | 128.0 | 0.0 |
| cPTSD | TSI_IA | 30.0 | 0.0 |
| cPTSD | TSI_IE | 29.0 | 0.0 |
| cPTSD | TSI_ISR | 25.0 | 0.0 |
| cPTSD | TSI_SOM | 27.0 | 0.0 |
| cPTSD | TSI_SUI | 20.0 | 0.0 |
| cPTSD | TSI_SXD | 20.0 | 0.0 |
| cPTSD | TSI_TRB | 25.0 | 0.0 |
| cPTSD | skPTSD_total_value | 184.0 | 66.0 |
| BPD | ACE_D | 8.0 | 0.0 |
| BPD | BDI | 51.0 | 6.0 |
| BPD | BSL_23 | 2.956521739130435 | 0.1739130434782609 |
| BPD | DERS_Emo_Aware | 29.0 | 6.0 |
| BPD | DERS_Emo_clarity | 25.0 | 7.0 |
| BPD | DERS_Goal_dir_Beh | 25.0 | 8.0 |
| BPD | DERS_Impulse_Control | 29.0 | 9.0 |
| BPD | DERS_Limit_Access_ER | 40.0 | 10.0 |
| BPD | DERS_Nonacceptance | 30.0 | 7.0 |
| BPD | DERS_total | 177.0 | 65.0 |
| BPD | FDS_20 | 47.5 | 0.0 |
| BPD | FIS_Responsibility_Guilt | 33.0 | 12.0 |
| BPD | FIS_Separation_Guilt | 26.0 | 8.0 |
| BPD | FIS_Survival_Guilt | 35.0 | 7.0 |
| BPD | FIS_total | 91.0 | 27.0 |
| BPD | GAD_7 | 18.0 | 2.0 |
| BPD | LEC_5 | 99.0 | 0.0 |
| BPD | OPD_Interpersonal_Contact | 16.0 | 3.0 |
| BPD | OPD_Relationship_Models | 16.0 | 0.0 |
| BPD | OPD_SF_Total | 44.0 | 4.0 |
| BPD | OPD_Self_Perception | 15.0 | 1.0 |
| BPD | PDS_Funct_Impair | 9.0 | 0.0 |
| BPD | PDS_Severity | 31.0 | 0.0 |
| BPD | PHQ_D | 23.0 | 6.0 |
| BPD | SF12_Mental_Summary | 50.81335 | 11.82 |
| BPD | SF12_Physical_Summary | 66.67 | 32.35 |
| BPD | TEPS_ANT | 58.0 | 20.0 |
| BPD | TEPS_CON | 44.0 | 14.0 |
| BPD | TEPS_Total | 102.0 | 30.0 |
| BPD | TSI_AA | 27.0 | 6.0 |
| BPD | TSI_ANG | 26.0 | 2.0 |
| BPD | TSI_D | 29.0 | 0.0 |
| BPD | TSI_DA | 28.0 | 0.0 |
| BPD | TSI_DIS | 19.0 | 0.0 |
| BPD | TSI_F_Ext | 84.0 | 6.0 |
| BPD | TSI_F_Self | 85.0 | 6.0 |
| BPD | TSI_F_Soma | 23.0 | 0.0 |
| BPD | TSI_F_Trauma | 90.0 | 12.0 |
| BPD | TSI_IA | 30.0 | 4.0 |
| BPD | TSI_IE | 25.0 | 0.0 |
| BPD | TSI_ISR | 29.0 | 0.0 |
| BPD | TSI_SOM | 23.0 | 0.0 |
| BPD | TSI_SUI | 25.0 | 0.0 |
| BPD | TSI_SXD | 24.0 | 0.0 |
| BPD | TSI_TRB | 29.0 | 1.0 |
| BPD | skPTSD_total_value | 184.0 | 0.0 |

Note: Minimum and maximum observed values per variable in each group.

**Table S7: BDI Proportions**

| Group | BDI_cat | n | prop |
| --- | --- | --- | --- |
| CON | Mild | 2 | 0.05263157894736842 |
| CON | Minimal | 36 | 0.9473684210526315 |
| cPTSD | Mild | 2 | 0.05882352941176471 |
| cPTSD | Moderate | 9 | 0.2647058823529412 |
| cPTSD | Severe | 23 | 0.6764705882352942 |
| BPD | Mild | 4 | 0.16 |
| BPD | Minimal | 3 | 0.12 |
| BPD | Moderate | 7 | 0.28 |
| BPD | Severe | 11 | 0.44 |

Note: BDI cutoff: Minimal (0-13), Mild (14-19), Moderate (20-28), Severe (≥29).

**Table S8: PHQ Proportions**

| Group | PHQ_cat | n | prop |
| --- | --- | --- | --- |
| CON | Mild | 11 | 0.2894736842105263 |
| CON | Minimal | 27 | 0.7105263157894737 |
| cPTSD | Mild | 2 | 0.05882352941176471 |
| cPTSD | Minimal | 1 | 0.02941176470588235 |
| cPTSD | Moderate | 10 | 0.2941176470588235 |
| cPTSD | Moderately severe | 9 | 0.2647058823529412 |
| cPTSD | Severe | 12 | 0.3529411764705883 |
| BPD | Mild | 7 | 0.28 |
| BPD | Moderate | 9 | 0.36 |
| BPD | Moderately severe | 8 | 0.32 |
| BPD | Severe | 1 | 0.04 |

Note: PHQ-9 cutoff: Minimal (0-4), Mild (5-9), Moderate (10-14), Moderately severe (15-19), Severe (≥20).

**Table S9: GAD Proportions**

| Group | GAD_cat | n | prop |
| --- | --- | --- | --- |
| CON | Mild | 3 | 0.07894736842105263 |
| CON | Minimal | 34 | 0.8947368421052632 |
| CON | Moderate | 1 | 0.02631578947368421 |
| cPTSD | Mild | 12 | 0.3529411764705883 |
| cPTSD | Minimal | 1 | 0.02941176470588235 |
| cPTSD | Moderate | 11 | 0.3235294117647059 |
| cPTSD | Severe | 10 | 0.2941176470588235 |
| BPD | Mild | 10 | 0.4 |
| BPD | Minimal | 2 | 0.08 |
| BPD | Moderate | 7 | 0.28 |
| BPD | Severe | 6 | 0.24 |

Note: GAD-7 cutoff: Minimal (0-4), Mild (5-9), Moderate (10-14), Severe (≥15).

**Table S10: ACE Proportions**

| Group | ACE_cat | n | prop |
| --- | --- | --- | --- |
| CON | <4 | 35 | 0.9210526315789473 |
| CON | High risk (>=4) | 3 | 0.07894736842105263 |
| cPTSD | <4 | 7 | 0.2058823529411765 |
| cPTSD | High risk (>=4) | 27 | 0.7941176470588235 |
| BPD | <4 | 9 | 0.36 |
| BPD | High risk (>=4) | 16 | 0.64 |

Note: ACE-D cutoff: High risk defined as score ≥4.

**Table S11: FDS Proportions**

| Group | FDS_cat | n | prop |
| --- | --- | --- | --- |
| CON | Below cutoff | 38 | 1.0 |
| cPTSD | Above cutoff | 29 | 0.8529411764705882 |
| cPTSD | Below cutoff | 4 | 0.1176470588235294 |
| cPTSD | nan | 1 | 0.02941176470588235 |
| BPD | Above cutoff | 15 | 0.6 |
| BPD | Below cutoff | 10 | 0.4 |

Note: FDS-20 cutoff: Score ≥13 indicates clinically relevant dissociation.

**Table S12: BSL-23 Proportions**

| Group | BSL_cat | n | prop |
| --- | --- | --- | --- |
| CON | Mild | 6 | 0.1578947368421053 |
| CON | Moderate | 2 | 0.05263157894736842 |
| CON | None/low | 30 | 0.7894736842105263 |
| cPTSD | High | 11 | 0.3235294117647059 |
| cPTSD | Mild | 2 | 0.05882352941176471 |
| cPTSD | Moderate | 10 | 0.2941176470588235 |
| cPTSD | None/low | 8 | 0.2352941176470588 |
| cPTSD | Very high | 3 | 0.08823529411764706 |
| BPD | High | 5 | 0.2 |
| BPD | Mild | 1 | 0.04 |
| BPD | Moderate | 15 | 0.6 |
| BPD | None/low | 1 | 0.04 |
| BPD | Very high | 3 | 0.12 |

Note: BSL-23 cutoff: None/low (<0.3), Mild (0.3-0.6), Moderate (0.7-1.6), High (1.7-2.6), Very high (2.7-3.4), Extremely high (≥3.5).

**Table S13: Internal Consistencies**

| **Scale** | **Subscale** | **Alpha** |
| --- | --- | --- |
| PHQ-D | Total | 0,915 |
| BDI-II | Total | 0,957 |
| GAD-7 | Total | 0,915 |
| DERS | Nonacceptance | 0,898 |
| DERS | Goals | 0,873 |
| DERS | Impulse | 0,759 |
| DERS | Awareness | 0,856 |
| DERS | Strategies | 0,943 |
| DERS | Clarity | 0,835 |
| DERS | Total | 0,976 |
| OPD-SF | Total | 0,941 |
| FDS-20 | Total | 0,948 |
| ACE-D | Total | 0,853 |
| TEPS | Anticipatory | 0,76 |
| TEPS | Consummatory | 0,786 |
| TEPS | Total | 0,876 |
| FIS | SurvivalGuilt | 0,909 |
| FIS | SeparationGuilt | 0,709 |
| FIS | ResponsibilityGuilt | 0,819 |
| FIS | Total | 0,912 |
| BSL-23 | Total | 0,959 |
| PDS | severity | 0,958 |
| PDS | impair | 0,908 |
| PDS | Total | 0,957 |

Note: Cronbach’s alpha values are reported for each scale and subscale based on the present sample. Internal consistencies above .70 are generally considered acceptable, values above .80 good, and values above .90 excellent.
